# Levosimendan reduces segmental pulmonary vascular resistance in isolated perfused rat lungs and relaxes human pulmonary vessels

**DOI:** 10.1371/journal.pone.0233176

**Published:** 2020-05-18

**Authors:** Annette Dorothea Rieg, Said Suleiman, Nina Andrea Bünting, Eva Verjans, Jan Spillner, Heike Schnöring, Sebastian Kalverkamp, Thomas Schröder, Saskia von Stillfried, Till Braunschweig, Gereon Schälte, Stefan Uhlig, Christian Martin

**Affiliations:** 1 Department of Anaesthesiology, Medical Faculty Aachen, Rhenish Westphalian Technical University, Aachen, Germany; 2 Institute of Pharmacology and Toxicology, Medical Faculty Aachen, Rhenish Westphalian Technical University, Aachen, Germany; 3 Department of Paediatrics, Medical Faculty Aachen, Rhenish Westphalian Technical University, Aachen, Germany; 4 Department of Cardiac and Thoracic Surgery, Medical Faculty Aachen, Rhenish-Westphalian Technical University, Aachen, Germany; 5 Department of Surgery, Luisenhospital Aachen, Aachen, Germany; 6 Institute of Pathology, Medical Faculty Aachen, Rhenish-Westphalian Technical University, Aachen, Germany; University of Mississippi Medical Center, UNITED STATES

## Abstract

**Introduction:**

Levosimendan is approved for acute heart failure. Within this context, pulmonary hypertension represents a frequent co-morbidity. Hence, the effects of levosimendan on segmental pulmonary vascular resistance (PVR) are relevant. So far, this issue has been not studied. Beyond that the relaxant effects of levosimendan in human pulmonary vessel are unknown. We addressed these topics in rats’ isolated perfused lungs (IPL) and human precision-cut lung slices (PCLS).

**Material and methods:**

In IPL, levosimendan (10 μM) was perfused in untreated and endothelin-1 pre-contracted lungs. The pulmonary arterial pressure (P_PA_) was continuously recorded and the capillary pressure (P_cap_) was determined by the double-occlusion method. Thereafter, segmental PVR, expressed as precapillary (R_pre_) and postcapillary resistance (R_post_) and PVR were calculated. Human PCLS were prepared from patients undergoing lobectomy. Levosimendan-induced relaxation was studied in naïve and endothelin-1 pre-contracted PAs and PVs. In endothelin-1 pre-contracted PAs, the role of K^+^-channels was studied by inhibition of K_ATP_-channels (glibenclamide), BK_Ca_^2+^-channels (iberiotoxin) and K_v_-channels (4-aminopyridine). All changes of the vascular tone were measured by videomicroscopy. In addition, the increase of cAMP/GMP due to levosimendan was measured by ELISA.

**Results:**

Levosimendan did not relax untreated lungs or naïve PAs and PVs. In IPL, levosimendan attenuated the endothelin-1 induced increase of P_PA_, PVR, R_pre_ and R_post_. In human PCLS, levosimendan relaxed pre-contracted PAs or PVs to 137% or 127%, respectively. In pre-contracted PAs, the relaxant effect of levosimendan was reduced, if K_ATP_- and K_v_-channels were inhibited. Further, levosimendan increased cGMP in PAs/PVs, but cAMP only in PVs.

**Discussion:**

Levosimendan reduces rats’ segmental PVR and relaxes human PAs or PVs, if the pulmonary vascular tone is enhanced by endothelin-1. Regarding levosimendan-induced relaxation, the activation of K_ATP_- and K_v_-channels is of impact, as well as the formation of cAMP and cGMP. In conclusion, our results suggest that levosimendan improves pulmonary haemodynamics, if PVR is increased as it is the case in pulmonary hypertension.

## Introduction

The Ca^2+^-sensitizer levosimendan exerts beneficial cardiovascular properties [1], decreases patients’ mortality in acute heart failure [2] and improves the outcome of cardiac surgical patients with left ventricular dysfunction [3,4]. Recently, levosimendan even convinced in outpatients, as the intermittent intravenous application of levosimendan reduced their hospitalization due to heart failure [5].

Beyond the inotropic effects of levosimendan, its dilating properties on the pulmonary circulation are of considerable impact [6–8]. They contribute to the success of levosimendan within the therapy of right heart failure and pulmonary arterial hypertension (PAH) [9–13]. However, studies targeting the relaxant effect of levosimendan in the pulmonary vascular bed are scare and most of them addressed pulmonary arteries (PAs) [6,8,11,14]. Although, relaxation of pulmonary veins (PVs) would be beneficial within pulmonary hypertension (PH) due to left-heart disease (LHD), which is the most common cause of PH [15] and primarily affects the pulmonary venous bed [16–18]. Recently, our group addressed this topic and studied the relaxant effects of levosimendan in central PAs and PVs using precision-cut lung slices (PCLS) of guinea pigs [8]. We found that levosimendan relaxes PAs and PVs via common (K_ATP_-channels, cAMP/cGMP) and different (BK_Ca_^2+^-and K_v_-channels only in PAs) mechanisms. Although, this study proved the relaxant effect of levosimendan in PAs and PVs and further illustrates that both vessel types respond different to the same stimulus [19,20], it has several restrictions. First of all, we studied PAs and PVs deriving from a central part of the lung which primarily do not determine pulmonary vascular resistance (PVR) [21,22]. Thus, with regard to PVR and particularly with regard to segmental PVR (precapillary (R_pre_) and postcapillary resistance (R_post_)), the effects of levosimendan remain unknown. Second, disregarding from PAs or PVs, the various segments along the pulmonary bed react quite different to various pharmacological stimuli [20]. So far, it is unexplored, whether levosimendan also exerts relaxation in a more peripheral part of the lung. Third, remarkable differences exist between various species [23–25]; hence we do not know if levosimendan-induced relaxation is also relevant for human PAs and PVs.

To address these items, we applied levosimendan in isolated perfused lungs (IPL) of rats using endothelin-1 (ET-1) to increase PVR. Further, we evaluated the relaxant effect of levosimendan in human PAs or PVs which derive from a peripheral part of the lung, as well as its effect on the formation of cAMP and cGMP. In addition, we studied the role of K_ATP_-, K_v_- and BK_Ca_^2+^-channels within levosimendan-induced relaxation in human PAs (PCLS).

## Material and methods

### Animals and human lung tissue

Female Wistar rats (250 ± 50 g) were purchased from Charles River (Sulzfeld, Germany). All animal studies were approved by the Landesamt für Natur, Umwelt und Verbraucherschutz Nordrhein-Westfalen (ID: 8.87–51.05.20.10.245) and all experiments were strictly performed due to the Directive 2010/63/EU of the European Parliament.

Human PCLS were prepared from patients undergoing lobectomy due to lung cancer. After pathological inspection, cancer free tissue from a peripheral part of the lung was used. Patients with PH (histology) were excluded. The study was approved by the local ethics committee (EK 61/09) of the Medical Faculty Aachen, Rhenish-Westphalian Technical University (RWTH) Aachen and all experiments were performed according to the Declaration of Helsinki. All patients gave written informed consent.

### Isolated perfused rat lungs

Rat lungs were prepared as described [26,27]. Briefly, intraperitoneal anaesthesia was performed (pentobarbital: 95 mg kg^-1^) and verified by missing reflexes. The rat was bled, the trachea cannulated and the lung ventilated with positive pressure (70 breaths/min). The apex of the left ventricle was cut and cannulas were placed in the pulmonary artery (perfusion inflow) and in the left atrium (perfusion outflow). The lung was perfused at constant flow (12,5 mL/min) with Krebs-Henseleit buffer, containing 2% bovine serum albumin, 0.1% glucose, 0.3% HEPES and 50 nM salbutamol to prevent bronchoconstriction [28]. The temperature of the perfusate was maintained at 37°C with a water bath and the pH was adjusted between 7.35 and 7.45 by carbon dioxide gassing. Heart and lungs were removed and set into a negative-pressure chamber. To prevent the formation of lung oedema during constant flow perfusion and negative pressure ventilation, a pressure balancing chamber was established in the perfusion outflow which was connected to the artificial thorax chamber. To prevent atelectasis of the lung, every 5 minutes a deep breath was applied. The tidal volume (TV), compliance (Cdyn), resistance (Res), pulmonary arterial pressure (P_PA_), pulmonary atrial pressure (P_LA_) and the flow were continuously monitored. As soon as respiratory and haemodynamic variables remained stable over 10 minutes, ET-1 (final concentration in the buffer: 15 nM) was added to the recirculating perfusion buffer (total volume 200 mL) to enhance PVR which was calculated as followed: PVR = (P_PA_−P_LA_) x 80 / flow. Ten minutes after the application of ET-1, levosimendan (10 μM) was perfused. Thereafter, changes of P_cap_ were measured every 10 minutes by the double-occlusion method (DOC) [27]. Therefore, two magnet valves mounted before and after the lung were simultaneously clamped to interrupt the perfusion in-/outflow at the same time. R_post_ and R_pre_ were calculated as followed: ${\text{R}}_{\text{post}}=\frac{Pcap-PLA}{flow}$ and ${\text{R}}_{\text{pre}}=\frac{PPA-Pcap}{flow}$.

### PCLS

Human lung lobes were filled via a main bronchus with 1.5% low-melting agarose and cooled on ice. Tissue cores were prepared and cut into approximately 300 μm thick slices with a tissue slicer (Alabama Research & Development, Munford, AL, USA). PCLS were incubated at 37°C and the medium was changed several times in order to wash out the agarose. PCLS are known to be at least 72 h viable [29,30].

### Measurements of cAMP and cGMP

To analyse cAMP and cGMP, human PAs and PVs from tissue cores were cannulated by a plastic catheter (14 gauges), isolated, flushed with levosimendan (100 μM and 1 μM) or medium (controls) and incubated for 30 minutes in medium. Afterwards, they were frozen by liquid N_2_. Cyclic AMP and cGMP were quantified with ELISA-kits following the manufacturer’s protocol. For stabilisation, all samples and standards were acetylated. To measure cAMP, all samples were diluted 1:2 with 0.1 M HCL. Both ELISAs were evaluated at 405 nM (GENIOS, Tecan, Switzerland).

### Identification of the vessels, histology

Human pulmonary vessels were identified by their anatomical landmarks. PAs accompany the airways and PVs lie aside. After the experiments, the identification of the vessels was histologically confirmed by elastica van Gieson-staining, where PAs show an internal and external elastic lamina, in contrast to PVs which show only an external elastic lamina [31].

### Measurements and imaging

The kinetics of levosimendan was studied. Human PCLS were exposed 5 minutes to each concentration of levosimendan. If pre-constriction was required, they were pre-treated 1h with ET-1. If K^+^-channels were inhibited, PCLS were pre-treated 1 h with 10 μM glibenclamide (K_ATP_-channels), 100 nM iberiotoxin (BK_Ca_^2+^-channels) or 5 mM 4-aminopyridine (K_v_-channels). If both were required, PCLS were exposed at once to both. Before the measurements, the initial vessel area (IVA) was defined as 100% and any relaxant or contractile effect (ET-1) was indicated as “Change of IVA [%]”. To compare relaxation of pre-treated vessels, the vessel area was defined after the pre-treatment duration of 1 h again as 100%. Hence, a vessel area <100% indicates contraction and a vessel area >100% indicates relaxation. Concentration-response curves of levosimendan were performed and the effects were indicated again as “Change of IVA [%]”. Control experiments were performed on consecutive sections. Pulmonary vessels were imaged by a digital video camera (Leica Viscam 1280 or Leica DFC 280). The images were analysed with Optimas 6.5 (Media Cybernetics, Bothell, WA) [29].

### Agents and culture medium

All agents were bought from Tocris Bioscience (Ellisville, Missouri, USA), except levosimendan which was from Sigma-Aldrich (Steinheim, Germany) and ET-1 which was from BIOTRENDS (Wangen, Switzerland).

### Statistics

**Fig 1:**
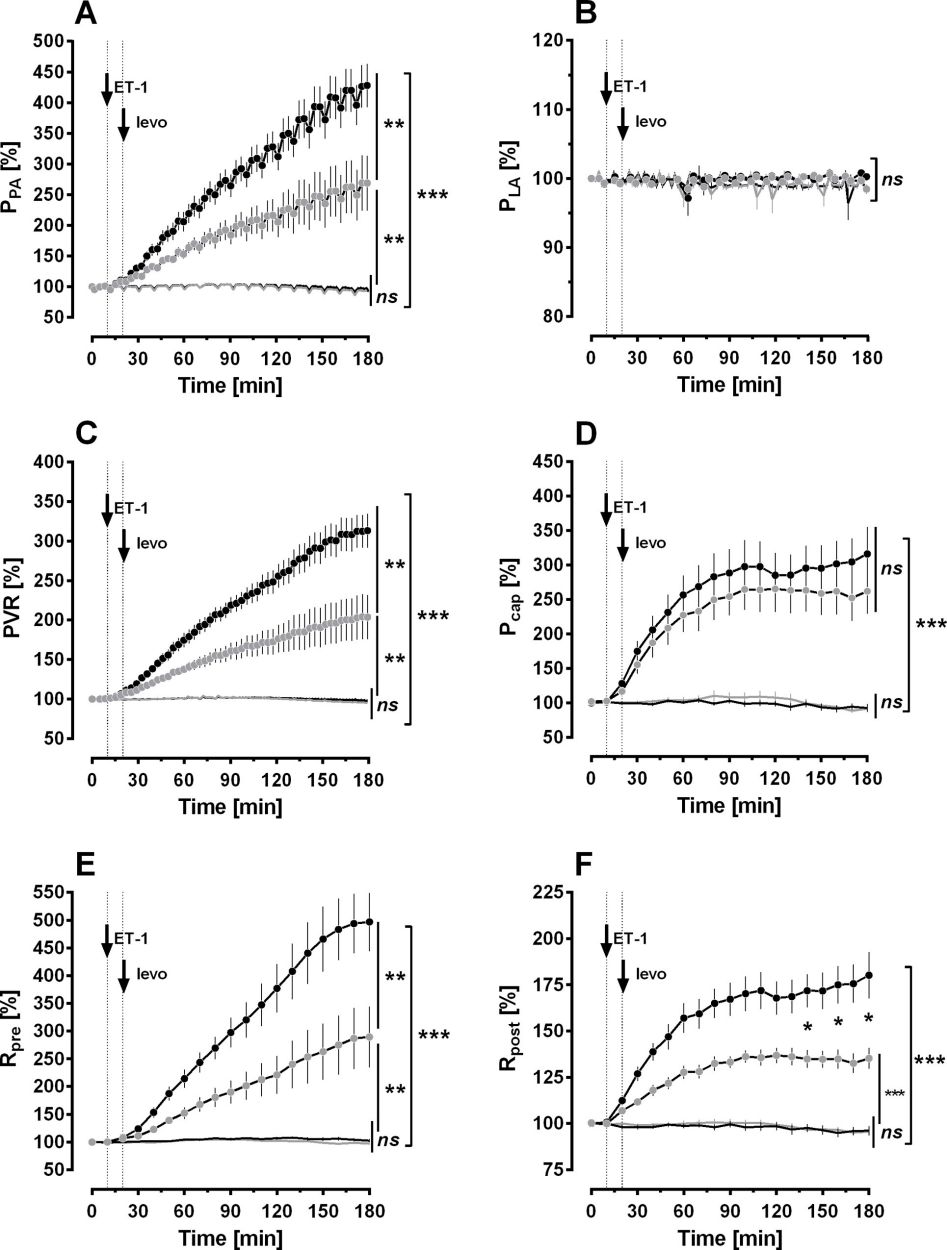
Effect of ET-1 and levosimendan on pulmonary haemodynamics in the IPL. **A) Pulmonary arterial pressure (P_PA_). B) Left atrial pressure (P_LA_). C) Pulmonary vascular resistance (PVR). D) Pulmonary capillary pressure (P_cap_). E) Precapillary resistance (R_pre_). F) Postcapillary resistance (R_post_). For all: ▔** control (n = 8); ▔ levosimendan (n = 8); ● 15 nM ET-1 (n = 7); ● 15 nM ET-1 / 10 μM levosimendan (n = 8). **A-F:** Statistics was performed by a linear mixed model analysis (LMM). P<0.05 are considered as significant: * p<0.05, ** p<0.01 and *** p<0.001.

**Fig 2:**
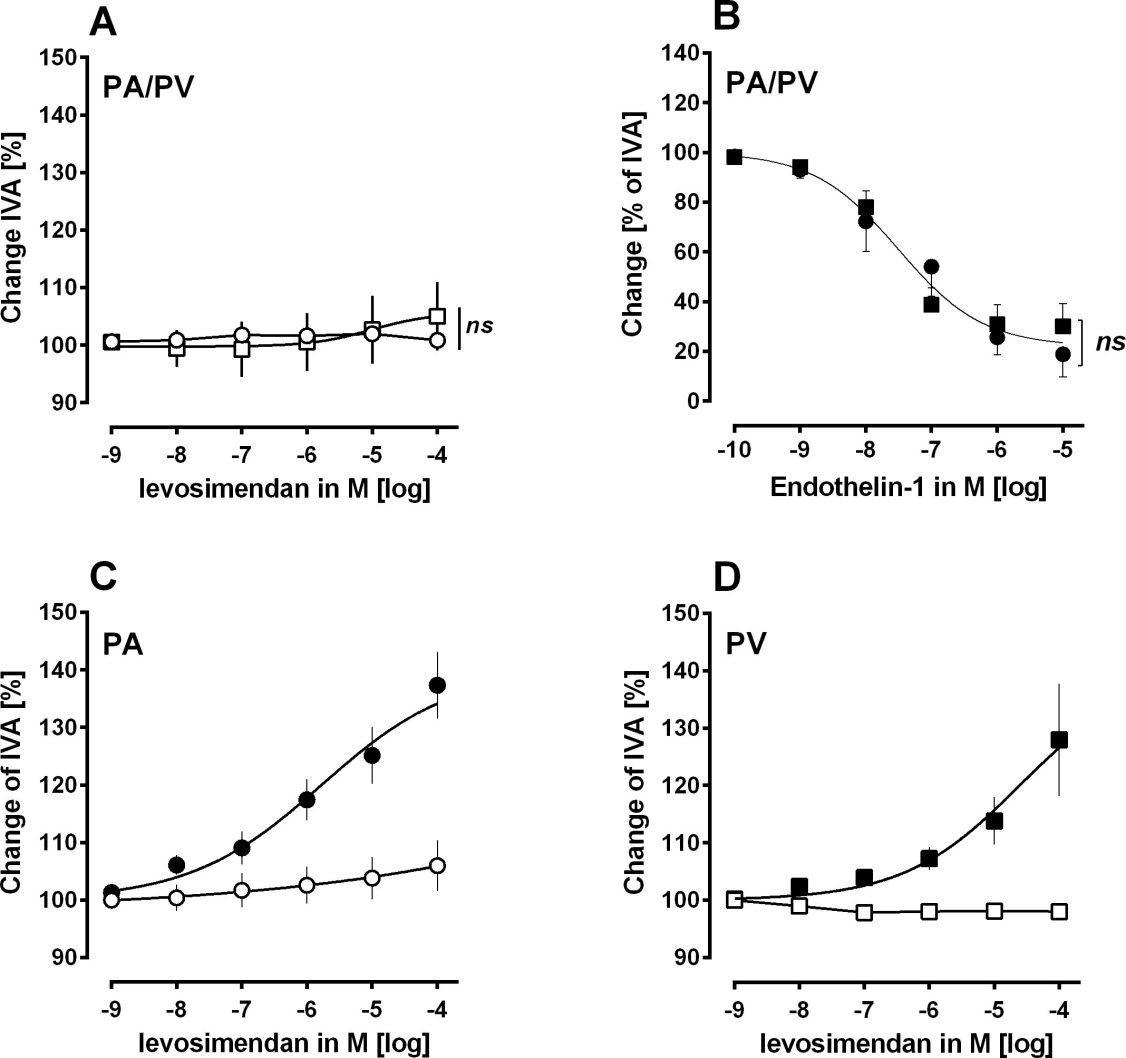
Effect of ET-1 and levosimendan in human PCLS. **A) Concentration-response curve of levosimendan in PAs and PVs:** ○ naïve PAs (n = 5); □ naïve PVs (n = 6). **B) Concentration-response curve of ET-1:** ● naïve PAs (n = 7); ■ naïve PVs (n = 6). **C) Concentration-response curve of levosimendan in ET-1 pre-constricted PAs:** ○ 100 nM ET-1 (n = 6); ● 100 nM ET-1 / levosimendan (n = 6). **D) Concentration-response curve of levosimendan in ET-1 pre-constricted PVs:** □ 50 nM ET-1 (n = 7); ■ 50 nM ET-1 / levosimendan (n = 8). **B:** Statistics was performed by the comparison of EC_50_ values. P<0.05 are considered as significant.

**Fig 3:**
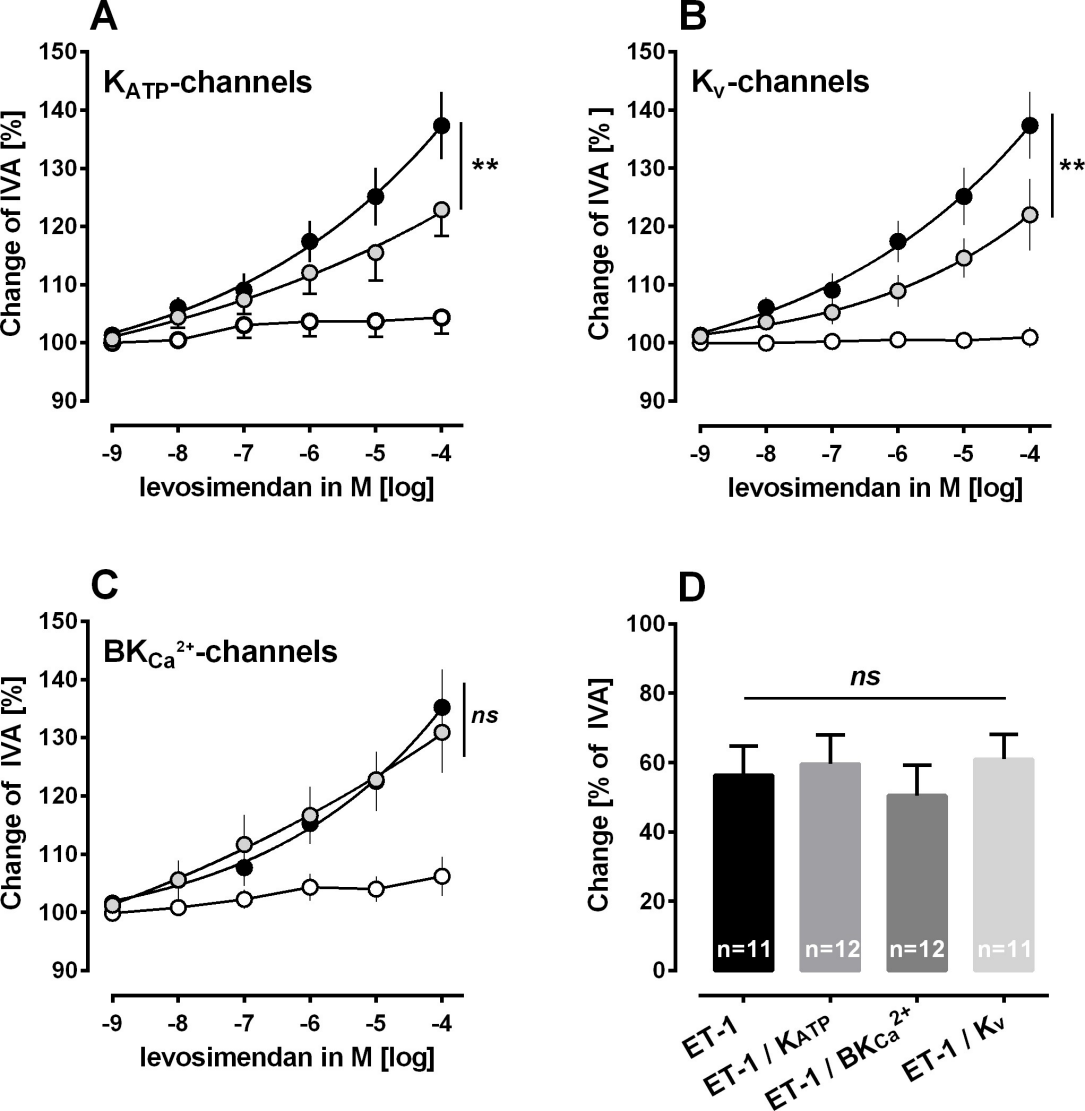
Role of K^+^-channels within the relaxant effect of levosimendan in human PCLS. **A) Inhibition of K**_**ATP**_**-channels (glibenclamide) in ET-1 pre-constricted PAs:** ○ 100 nM ET-1 / 10 μM glibenclamide (n = 6); ● 100 nM ET-1 / 10 μM glibenclamide / levosimendan (n = 6); ● 100 nM ET-1 / levosimendan (n = 6). **B) Inhibition of K**_**V**_**-channels (5-aminopyridine) in ET-1 pre-constricted PAs:** ○ 100 nM ET-1 / 5 mM 5-aminopyridine (n = 6); ● 100 nM ET-1 / 5 mM 5-aminopyridine / levosimendan (n = 6); ● 100 nM ET-1 / levosimendan (n = 6). **C) Inhibition of BK**_**Ca**_^**2+**^**-channels (iberiotoxin) in ET-1 pre-constricted PAs:** ○ 100 nM ET-1 / 100 nM iberiotoxin (n = 5); ● 100 nM ET-1 / 100 nM iberiotoxin / levosimendan (n = 5s); ● 100 nM ET-1 / levosimendan (n = 5). **D) Effect of inhibition of K**^**+**^**-channels on the contractile effect of ET-1. A-C:** Statistics was performed by the comparison of EC_50_ values. **D:** Statistic was performed by an one-way ANOVA. P<0.05 are considered as significant: * p<0.05, ** p<0.01 and *** p<0.001.

**Fig 4:**
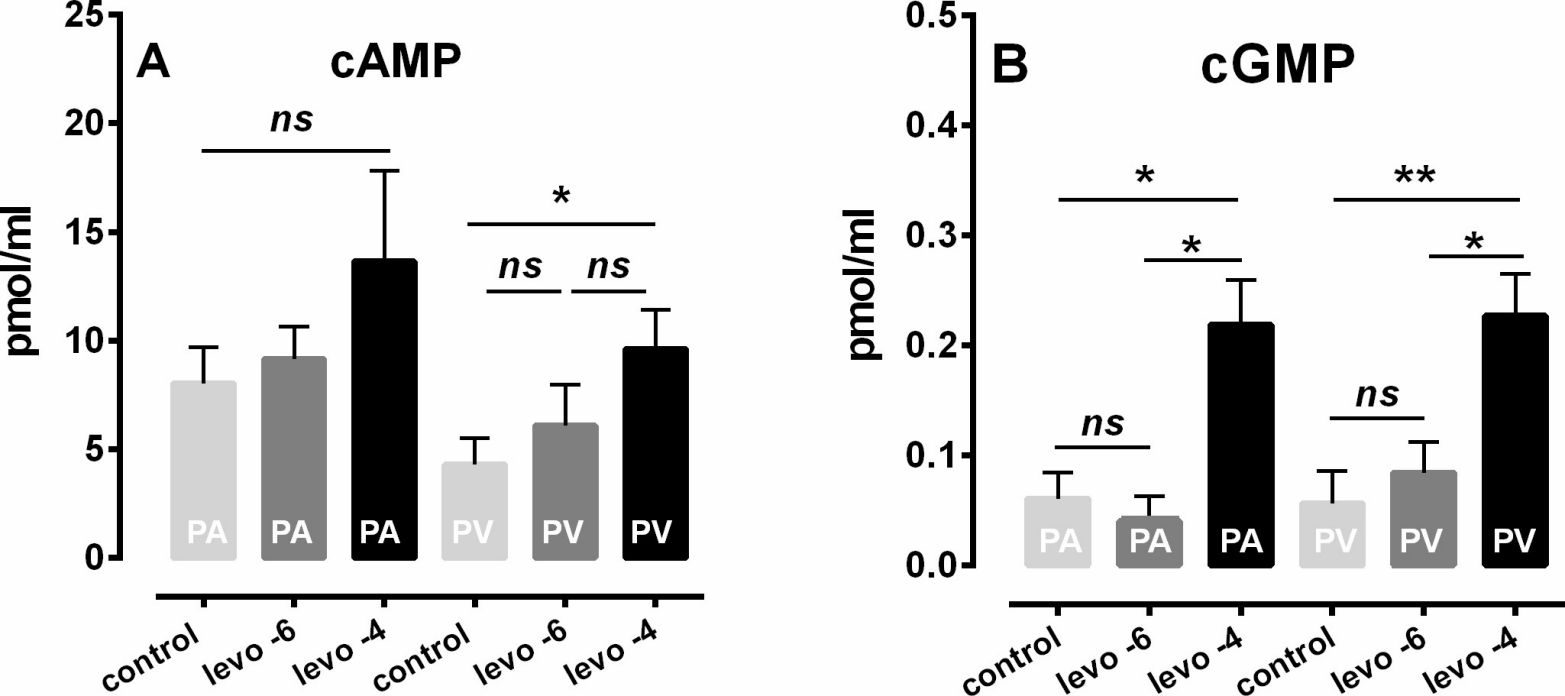
Effects of levosimendan on intracellular cAMP and cGMP in human PAs and PVs. **A): cAMP:** ■ PA: control (n = 5); ■ PA: 1 μM levosimendan (n = 4); ■ PA: 100 μM levosimendan (n = 7); ■ PV: control (n = 6); ■ PV: 1 μM levosimendan (n = 4); ■ PV: 100 μM levosimendan (n = 8). **B): cGMP:** ■ PA: control (n = 5); ■ PA: 1 μM levosimendan (n = 3); ■ PA: 100 μM levosimendan (n = 6); ■ PV: control (n = 3); ■ PV: 1 μM levosimendan (n = 3); ■ PV: 100 μM levosimendan (n = 6). **A/B:** Statistics was performed by the Mann-Whitney U test. P<0.05 are considered as significant: * p<0.05 or ** p<0.01.

Statistics was conducted using SAS software 9.3 (SAS Institute, Cary, North Carolina, USA) and GraphPad Prism 5.01 (GraphPad, La Jolla, USA). The data in Fig 1 were analysed using a linear mixed model analysis (LMM) with variance components (VC) for the covariance matrix; EC_50_ values were calculated by the standard 4-paramter logistic non-linear regression model (Figs 2 and 3A–3C). The AIC-criterion was used to select the most parsimonious model, i.e. a common bottom, top, slope and EC_50_ value in the regression model or the covariance matrix with the least number of parameters. The data in Fig 3D were evaluated by a one-way ANOVA and the data in Fig 4 were analysed by the Mann-Whitney U test. P-values were adjusted for multiple comparisons by the false discovery rate and presented as mean ± SEM. P < 0.05 was considered as significant; (n) indicates the numbers of animals or lung lobes.

## Results

We studied the Ca^2+^-sensitizer levosimendan in the IPL using untreated and ET-1 pre-treated rat lungs. In this view, we focused on the pulmonary vascular properties of levosimendan. In human PCLS, we evaluated the impact of levosimendan-induced relaxation in PAs and PVs. In addition, we studied the impact of K^+^-channels within the relaxant effect of levosimendan in human PAs.

### Rat IPL: Effect of levosimendan on pulmonary haemodynamics

In the IPL, perfusion with 10 μM levosimendan was started, if baseline values of all parameters were stable for 20 minutes. Pulmonary haemodynamic parameters such as P_PA_, P_LA_, PVR, capillary pressure (P_cap_), R_pre_ and R_post_ remained stable during perfusion with levosimendan and did neither differ from baseline values nor from those of untreated control IPLs (Fig 1A–1F; p>0.05).

Next, we studied the effects of levosimendan in ET-1 pre-treated IPLs to increase PVR, as a pathophysiological feature of PH. Perfusion of ET-1 (final concentration in the buffer 15 nM) was started, if baseline parameters were stable for 10 minutes. ET-1 significantly increased P_PA_, PVR, P_cap_, R_pre_ and R_post_ (Fig 1A and 1C–1F; p<0.001), whereas P_LA_ remained unchanged (Fig 1B). Perfusion of levosimendan decreased the ET-1 induced increase of P_PA_, PVR, R_pre_ (Fig 1A/C/E; all: p<0.01) and R_post_ (Fig 1F; for 140–180 minutes: p<0.05). Even though, values of all parameters stayed higher than those of untreated control lungs, e.g. P_PA_, PVR, R_pre_ (Fig 1A 1C and 1E; all: p<0.01) and R_post_ (Fig 1F; p<0.001). In addition, levosimendan did not alter the effect of ET-1 on P_cap_ (Fig 1D).

### Human PCLS: The relaxant effect of levosimendan in human pulmonary arteries and veins

In human PCLS, we studied the pulmonary vasorelaxant properties of increasing concentrations of levosimendan. Levosimendan did not relax naïve PAs or PVs (Fig 2A). Thus, we decided to pre-contract PAs and PVs with ET-1. To reach a comparable degree of pre-contraction, we treated both with increasing concentrations of ET-1 (Fig 2B). ET-1 contracted PAs and PVs with EC_50_ values of 58 nM and 21 nM, respectively (Fig 2B). Thus, we decided to pre-contract PAs and PVs with 100 nM and 50 nM ET-1, respectively. After pre-contraction, 100 μM levosimendan relaxed PAs up to 137% of IVA with an EC_50_ value of 1.6 μM (Fig 2C). Levosimendan at 1 μM was also effective, as it relaxed PAs up to 117% of IVA (Fig 2C). Further, 100 μM levosimendan relaxed PVs up to 127% of IVA with an EC_50_ value of 24 μM, whereas 1 μM levosimendan relaxed PVs only to 107% of IVA (Fig 2D). Finally, levosimendan relaxed humans PAs more potently than human PVs (p<0.05).

### Mechanisms contributing to the relaxant effect of levosimendan—K^+^-channels

Next, we studied the involvement of K^+^-channels within the relaxant effect of levosimendan. We studied this issue in human PAs, as they relaxed stronger to levosimendan than human PVs. Inhibition of K_ATP_-channels by glibenclamide decreased the relaxant effect of levosimendan in human PAs and shifted the EC_50_ value from 1.6 to 22 μM (Fig 3A; p<0.01). Inhibition of K_v_-channels by 4-aminopyridine also decreased levosimendan-induced relaxation, EC_50_ values were displaced rightwards from 1.6 μM to 56 μM (Fig 3B; p<0.01). In contrast, inhibition of BK_Ca_^2+^-channels did not alter the relaxant effect of levosimendan (Fig 3C). In addition, inhibition of K^+^-channels did not alter ET-1-induced contraction (Fig 3D).

### Mechanisms contributing to the relaxant effect of levosimendan—cAMP/cGMP

In human PAs, levosimendan did not enhance intracellular cAMP-levels, neither at 1 or 100 μM (Fig 4A). In contrast, 100 μM levosimendan increased intracellular cAMP in human PVs (Fig 4A; p<0.05), whereas a lower concentration of 1 μM levosimendan was without effect (Fig 4A). Further, levosimendan at 100 μM enhanced intracellular cGMP in PAs and PVs (Fig 4B; p<0.05 for PAs and p<0.01 for PVs), whereas levosimendan at 1 μM had no effect on cGMP levels.

## Discussion

In ET-1 pre-treated IPLs of the rat, we could prove the lowering effect of levosimendan on total PVR and on segmental PVR, expressed as R_pre_ and R_post_. Beyond that we could show that this effect is relevant for humans, as levosimendan relaxes ET-1 pre-constricted human PAs and PVs from the peripheral part of the lung. Regarding the pulmonary vasorelaxant effects of levosimendan the activation of K_ATP_- and K_v_-channels, as well as the generation of cAMP/cGMP are of impact.

### ET-1-induced pre-constriction

We selected ET-1 to enhance the vascular tone in rats lungs and in human PAs/PVs, as ET-1 plays a major role in PH, e.g. ET-1 receptors are up-regulated [32] and ET-1 levels are increased [33,34]. Aside its role as a potent vasoconstrictor, ET-1 mediates proliferation and migration of vascular cells and thus promotes vascular remodelling in PH [35].

### IPL: Pulmonary vascular effects of levosimendan

In the IPL of the rat, we show that levosimendan reduces the increasing effects of ET-1 on P_PA_, PVR, R_pre_ and R_post_. In contrast, levosimendan was without effect on pulmonary haemodynamics, if P_PA_, PVR, R_pre_ and R_post_ were not enhanced. Our results concerning the effects of levosimendan on P_PA_, resembling the central pulmonary arterial bed are in line with other experimental set-ups [6,36]. In isolated perfused feline lung lobes [6], levosimendan reduced the increasing effects of thromboxane on P_PA_ and in a porcine model of hypoxia-induced PH [36]; levosimendan lowered the hypoxic increase of mean P_PA_ [36]. They are further consistent with a previous work, where levosimendan relaxed central ET-1 pre-constricted PAs from guinea pigs [8]. Beyond that, the beneficial effects of levosimendan on P_PA_ have been proven in clinical studies with patients suffering to PAH [11,37,38]. In summary, these data confirm the relevance of levosimendan-induced relaxation in central PAs.

In spite of the promising data with levosimendan and its relaxant effect on the central pulmonary arterial bed, relaxation of PAs/PVs from the peripheral pulmonary vascular bed would be much more valuable, as due to its high cross-sectional area, it mainly determines PVR [21,22]. In the present study, levosimendan significantly decreased PVR in ET-1 pre-treated lungs. Further, in hypoxia-induced PH levosimendan lowered the hypoxic increase of PVR [36]. Hence, both results highly suggest that a relevant relaxant effect of levosimendan in peripheral PAs or PVs should also account for levosimendan-induced reduction of PVR. To highlight this issue, we applied the DOC and found that levosimendan decreases segmental arterial and venous PVR, expressed as R_pre_ and R_post_. Both results are of high clinical value, e.g. levosimendan-induced reduction of R_pre_ means that right ventricular afterload decreases which is advantageous in PAH [9–13]. In contrast, levosimendan-induced reduction of R_post_ is of clinical importance in PH due to LHD, as small PVs contributing to PVR relax to levosimendan. In principle, PH due to LHD, also called postcapillary PH resembles the most common cause of PH [15] which mainly affects the pulmonary venous system [16,18]. So far, the effects of levosimendan on R_post_ have been unexplored, as the access to the pulmonary venous bed is quite difficult. In vivo, pulmonary venous pressures must be calculated indirectly from the pulmonary capillary wedge pressure (PCWP) which reflects the pressure in the left atrium [39] or in large PVs [40], but not in small PVs which are aside small PAs mainly responsible for PVR [21,22]. In the IPL, we calculated R_post_ and R_pre_ after determination of P_cap_ by the DOC [27].

### PCLS: Pulmonary vasorelaxant effects of levosimendan

In PCLS, levosimendan relaxed small human PAs and PVs potently. Thus, our results from the IPL concerning the effect of levosimendan on R_pre_ and R_post_ are supported and the clinical relevance of levosimendan is confirmed.

Levosimendan relaxed human PAs stronger than PVs; hence the present results differ in part from those of a former work analysing the relaxant effect of levosimendan in PAs and PVs from guinea pigs [8]. There, levosimendan relaxed pre-constricted PAs or PVs from guinea pigs comparable, in addition it also relaxed untreated naïve PVs [8]. Reflecting possible reasons for the varying response of human PAs and PVs to levosimendan, the degree of pre-constriction should be considered. Though, human PAs and PVs were comparable pre-constricted with 100 nM and 50 nM ET-1, respectively, as shown in Fig 2B and in a former study [31]. Finally, the degree of pre-constriction should not account for the different relaxant response of human PAs and PVs. In principle, PAs and PVs differ within their anatomical structure [41]. So, it is not entirely surprising that they differ also in their reaction to various stimuli, as it was already shown for cardiovascular agents, NO or prostacyclin [19,42–44]. Indeed, levosimendan does not only exert different vascular responses in respect to the pulmonary arterial or venous belonging of the studied vessel, but also in the dependence to the species, e.g. ET-1 pre-contracted PAs and PVs from the peripheral part of the human lung did not relax comparable to levosimendan, in contrast ET-1 pre-contracted PAs and PVs from the central part of the guinea pig lung relaxed comparable to levosimendan [8]. Certainly, not only different species, but also different vessel sizes were studied. This is of impact, as in dependence of the vessel size and the pulmonary vascular segment; pulmonary vessels exert a certain K^+^-channel diversity [20] and are differently equipped with α_1_/β_1/2_-receptors [44]. The meaning of the studied species is supported by several studies, e.g. pulmonary vessels from humans and guinea pigs varied in their reaction to imatinib [23,24], PDGF-BB [24,45] or milrinone [31]. Finally, to obtain valuable information concerning the vasorelaxant effects of several vasodilators in the human pulmonary vascular bed, human PAs and PVs should be studied.

### PCLS: The role of K^+^-channels within the relaxant effect of levosimendan

In human PAs, we studied the role of K^+^-channels within the relaxant effect of levosimendan. We found that activation of K_ATP_- and K_v_-channels belongs to the mechanisms beyond, whereas activation of BK_Ca_^2+^ is without impact. Our results concerning the role of K_ATP_-channels within levosimendan-induced relaxation are in line with previous studies studying PAs and PVs from guinea pigs [8], human internal thoracic arteries [46], human portal veins [47] or feline lung lobes [6]. Accordingly, the impact of K_v_-channel-activation within the relaxant effect has been already shown in central PAs from guinea pigs [8] and in porcine coronary arteries [48]. In contrast, our results concerning BK_Ca_^2+^-channels are not in line with a former work in central PAs from guinea pigs [8]. There, K_ATP_-, BK_Ca_^2+^- and K_v_-channels have been shown to be involved within levosimendan-induced relaxation [8]. In the present work, we studied human PAs deriving from a peripheral part of the lung; thus resistance PAs which are only rarely equipped with BK_Ca_^2+^-channels [20]. In contrast, previously we studied central PAs from guinea pigs [8], thus conduit PAs which are densely equipped with BK_Ca_^2+^-channels [20]. Finally, the diverging results are explainable by the heterogeneity of K^+^-channels along the pulmonary vasculature [20]. Apart from that, levosimendan-induced activation of BK_Ca_^2+^-channels has been already shown in porcine coronary arteries [48] and human internal thoracic arteries [46]. Above all, levosimendan-induced activation of K^+^-channels does not only account for its vasorelaxant effects, but also alters vascular remodeling in PH [14]. Revermann et al. [14] showed that levosimendan-induced activation of K_ATP_-channels attenuates the pulmonary vascular remodeling due to monocrotaline in rats. This issue is of particular interest in PH, as decreased expression and/or activity of K^+^-channels resembles an important pathophysiological aspect of pulmonary vascular remodeling in PH [35]. Hence, levosimendan could fulfill two therapeutic requirements within the therapy of PH, 1) pulmonary vascular relaxation and 2) reversal of pulmonary vascular remodelling.

### Human PAs and PVs–the role of levosimendan-induced generation of cAMP and cGMP

Levosimendan did not relax naïve human PAs/PVs, although 100 μM levosimendan increased cGMP in human PAs/PVs and cAMP in human PVs. In contrast, a lower concentration of 1 μM levosimendan did not influence the generation of cAMP or cGMP. Due to the fact that naïve human PAs/PVs did not relax to 100 μM levosimendan, although 100 μM levosimendan increased cAMP/cGMP-levels, it is most likely that levosimendan-induced generation of cAMP/cGMP is not relevant for its relaxant effect in the human pulmonary circulation. This conclusion is reinforced by the fact that plasma levels of 100 μM levosimendan are not reached in humans [49].

A previous study in central PAs and PVs from guinea pigs [8] indicates a cGMP-raising potential of levosimendan [8], there inhibition of cGMP did not alter the relaxant effect of levosimendan. In contrast, studies in coronary vessels showed increased cGMP-levels due to levosimendan which were relevant for its relaxant potential [14]. Regarding the effect of levosimendan on cAMP, our results are in contrast to a previous study [8]. There, levosimendan raised cAMP in naïve central PAs/PVs from guinea pigs [8]. In PVs from guinea pigs, the cAMP-increase was crucial for levosimendan-induced relaxation [8]. Beyond that, the raising effect of levosimendan on cAMP was shown in porcine coronary arteries [50]. In principle, the effects of levosimendan on cAMP are explainable by its inhibiting effects on PDE-III [51] and its stimulating effects on adenyl cyclase [8]. Both have been shown to be expressed in the human vascular bed [52]. Anyway, our results concerning the effects of levosimendan on cAMP differ in PAs from humans and guinea pigs [8]. Possibly, the expression of PDE-III and adenyl cyclase differs in dependence to the studied species and the vascular segment.

## Conclusion

Levosimendan lowers R_pre_, R_post_, P_PA_ and PVR if the pulmonary vascular tone is enhanced by ET-1 resembling a major pathological aspect of PH. The impact of these results is enforced by the fact that levosimendan relaxes human pulmonary vessels at concentrations which are clinically reached in patients; e.g. plasma levels of 850 nM [49]. Taken into consideration that the flow resistance increases 16 fold, if the radius divides in half (Hagen-Poiseuille law), the presented vascular effects in human PAs/PVs should be also relevant for human PVR.

These findings suggest a beneficial effect of levosimendan in cardiac surgical patients with PH and also propose its application in non-surgical patients with PH.

## Supporting information

S1 Data (ZIP)

S2 Data (ZIP)

S3 Data (ZIP)

## References

[1] Farmakis D, Alvarez J, Gal TB, Brito D, Fedele F, Fonseca C, et al (2016) Levosimendan beyond inotropy and acute heart failure: Evidence of pleiotropic effects on the heart and other organs: An expert panel position paper. Int J Cardiol 222: 303–312. S0167-5273(16)31597-2 [pii]; 10.1016/j.ijcard.2016.07.202 27498374

[2] Landoni G, Biondi-Zoccai G, Greco M, Greco T, Bignami E, Morelli A, et al (2012) Effects of levosimendan on mortality and hospitalization. A meta-analysis of randomized controlled studies*. Crit Care Med 40: 634–646. 10.1097/CCM.0b013e318232962a 21963578

[3] Harrison RW, Hasselblad V, Mehta RH, Levin R, Harrington RA, Alexander JH (2013) Effect of levosimendan on survival and adverse events after cardiac surgery: a meta-analysis. J Cardiothorac Vasc Anesth 27: 1224–1232. S1053-0770(13)00171-7 [pii]; 10.1053/j.jvca.2013.03.027 24050857

[4] Lim JY, Deo SV, Rababa'h A, Altarabsheh SE, Cho YH, Hang D, et al (2015) Levosimendan Reduces Mortality in Adults with Left Ventricular Dysfunction Undergoing Cardiac Surgery: A Systematic Review and Meta-analysis. J Card Surg 30: 547–554. 10.1111/jocs.12562 25989324

[5] Comin-Colet J, Manito N, Segovia-Cubero J, Delgado J, Garcia Pinilla JM, Almenar L, et al (2018) Efficacy and safety of intermittent intravenous outpatient administration of levosimendan in patients with advanced heart failure: the LION-HEART multicentre randomised trial. Eur J Heart Fail. 10.1002/ejhf.1145 29405611

[6] De Witt BJ, Ibrahim IN, Bayer E, Fields AM, Richards TA, Banister RE, et al (2002) An analysis of responses to levosimendan in the pulmonary vascular bed of the cat. Anesth Analg 94: 1427–33. 10.1097/00000539-200206000-00009 12032000

[7] Kleber FX, Bollmann T, Borst MM, Costard-Jackle A, Ewert R, Kivikko M, et al (2009) Repetitive dosing of intravenous levosimendan improves pulmonary hemodynamics in patients with pulmonary hypertension: results of a pilot study. J Clin Pharmacol 49: 109–115. 10.1177/0091270008325150 18981240

[8] Rieg AD, Rossaint R, Verjans E, Maihofer NA, Uhlig S, Martin C (2013) Levosimendan Relaxes Pulmonary Arteries and Veins in Precision-Cut Lung Slices—The Role of K-Channels, cAMP and cGMP. PLoS ONE 8: e66195 10.1371/journal.pone.0066195;PONE-D-12-34289 [pii]. 23824760 PMC3688856

[9] Guerrero-Orriach JL, Ariza-Villanueva D, Florez-Vela A, Garrido-Sanchez L, Moreno-Cortes MI, Galan-Ortega M, et al (2016) Cardiac, renal, and neurological benefits of preoperative levosimendan administration in patients with right ventricular dysfunction and pulmonary hypertension undergoing cardiac surgery: evaluation with two biomarkers neutrophil gelatinase-associated lipocalin and neuronal enolase. Ther Clin Risk Manag 12: 623–630. 10.2147/TCRM.S102772;tcrm-12-623 [pii]. 27143905 PMC4844253

[10] Guerrero Orriach JL, Navarro A, I, Iglesias P, Galan OM, Rubio NM, Cruz MJ (2013) Preoperative levosimendan for right ventricular dysfunction before heart valve replacement surgery. Rev Esp Cardiol (Engl Ed) 66: 999–1000. S1885-5857(13)00183-7 [pii]; 10.1016/j.rec.2013.05.015 24774116

[11] Hansen MS, Andersen A, Holmboe S, Schultz JG, Ringgaard S, Simonsen U, et al (2017) Levosimendan Prevents and Reverts Right Ventricular Failure in Experimental Pulmonary Arterial Hypertension. J Cardiovasc Pharmacol 70: 232–238. 10.1097/FJC.0000000000000508 28640039

[12] Hansen MS, Andersen A, Nielsen-Kudsk JE (2018) Levosimendan in pulmonary hypertension and right heart failure. Pulm Circ 8: 2045894018790905. 10.1177/2045894018790905 29979110 PMC6058424

[13] Jiang R, Zhao QH, Wu WH, Zhang R, Yuan P, Gong SG, et al (2017) Efficacy and safety of a calcium sensitizer, levosimendan, in patients with right heart failure due to pulmonary hypertension. Clin Respir J. 10.1111/crj.12699 28862394

[14] Revermann M, Schloss M, Mieth A, Babelova A, Schroder K, Neofitidou S, et al (2011) Levosimendan attenuates pulmonary vascular remodeling. Intensive Care Med 37: 1368–1377. 10.1007/s00134-011-2254-9 21626431

[15] Guha A, Amione-Guerra J, Park MH (2016) Epidemiology of Pulmonary Hypertension in Left Heart Disease. Prog Cardiovasc Dis 59: 3–10. S0033-0620(16)30053-6 [pii]; 10.1016/j.pcad.2016.07.001 27402130

[16] Breitling S, Ravindran K, Goldenberg NM, Kuebler WM (2015) The pathophysiology of pulmonary hypertension in left heart disease. Am J Physiol Lung Cell Mol Physiol 309: L924–L941. ajplung.00146.2015 [pii]; 10.1152/ajplung.00146.2015 26502478

[17] Fayyaz AU, Edwards WD, Maleszewski JJ, Konik EA, DuBrock HM, Borlaug BA, et al (2018) Global Pulmonary Vascular Remodeling in Pulmonary Hypertension Associated With Heart Failure and Preserved or Reduced Ejection Fraction. Circulation 137: 1796–1810. CIRCULATIONAHA.117.031608 [pii]; 10.1161/CIRCULATIONAHA.117.031608 29246894 PMC5915920

[18] Schmeisser A, Schroetter H, Braun-Dulleaus RC (2013) Management of pulmonary hypertension in left heart disease. Ther Adv Cardiovasc Dis 7: 131–151. 1753944713477518 [pii]; 10.1177/1753944713477518 23592742

[19] Back M, Walch L, Norel X, Gascard JP, Mazmanian G, Brink C (2002) Modulation of vascular tone and reactivity by nitric oxide in porcine pulmonary arteries and veins. Acta Physiol Scand 174: 9–15. 10.1046/j.1365-201x.2002.00928.x 11851592

[20] Bonnet S, Archer SL (2007) Potassium channel diversity in the pulmonary arteries and pulmonary veins: implications for regulation of the pulmonary vasculature in health and during pulmonary hypertension. Pharmacol Ther 115: 56–69. S0163-7258(07)00076-9 [pii]; 10.1016/j.pharmthera.2007.03.014 17583356

[21] Gao Y, Raj JU (2005) Role of veins in regulation of pulmonary circulation. Am J Physiol Lung Cell Mol Physiol 288: L213–L226. 288/2/L213 [pii]; 10.1152/ajplung.00103.2004 15640520

[22] Raj JU, Ramanathan R, Chen P, Anderson J (1989) Effect of hematocrit on microvascular pressures in 3- to 5-wk-old rabbit lungs. Am J Physiol 256: H766–H771. 10.1152/ajpheart.1989.256.3.H766 2923236

[23] Maihofer NA, Suleiman S, Dreymuller D, Manley PW, Rossaint R, Uhlig S, et al (2017) Imatinib relaxes the pulmonary venous bed of guinea pigs. Respir Res 18: 32 10.1186/s12931-017-0514-0;10.1186/s12931-017-0514-0 [pii]. 28178968 PMC5299687

[24] Rieg AD, Bunting NA, Cranen C, Suleiman S, Spillner JW, Schnoring H, et al (2019) Tyrosine kinase inhibitors relax pulmonary arteries in human and murine precision-cut lung slices. Respir Res 20: 111 10.1186/s12931-019-1074-210.1186/s12931-019-1074-2 [pii]. 31170998 PMC6555704

[25] Schleputz M, Rieg AD, Seehase S, Spillner J, Perez-Bouza A, Braunschweig T, et al (2012) Neurally Mediated Airway Constriction in Human and Other Species: A Comparative Study Using Precision-Cut Lung Slices (PCLS). PLoS ONE 7: e47344 10.1371/journal.pone.0047344;PONE-D-12-11366 [pii]. 23056631 PMC3467211

[26] Rieg AD, Suleiman S, Perez-Bouza A, Braunschweig T, Spillner JW, Schroeder T, et al (2013) Milrinone relaxes pulmonary veins in guinea pigs and humans. PLoS One e87685. 10.1371/journal.pone.0087685 PMC3909212 24498166

[27] Uhlig S, Wollin L (1994) An improved setup for the isolated perfused rat lung. J Pharmacol Toxicol Methods 31: 85–94. 10.1016/1056-8719(94)90047-7 8032099

[28] Atzori L, Bannenberg G, Corriga AM, Moldeus P, Ryrfeldt A (1992) Sulfur dioxide-induced bronchoconstriction in the isolated perfused and ventilated guinea-pig lung. Respiration 59: 16–21. 10.1159/000196018 1579713

[29] Martin C, Uhlig S, Ullrich V (1996) Videomicroscopy of methacholine-induced contraction of individual airways in precision-cut lung slices. Eur Respir J 9: 2479–2487. 10.1183/09031936.96.09122479 8980957

[30] Ressmeyer A, Larsson A, Vollmer E, Dahlen S, Uhlig S, Martin C (2006) Characterisation of guinea pig precision-cut lung slices: comparison with human tissues. Eur Respir J 28: 603–611. 10.1183/09031936.06.00004206 16737991

[31] Rieg AD, Suleiman S, Perez-Bouza A, Braunschweig T, Spillner JW, Schroder T, et al (2014) Milrinone relaxes pulmonary veins in guinea pigs and humans. PLoS ONE 9: e87685 10.1371/journal.pone.0087685;PONE-D-13-18927 [pii]. 24498166 PMC3909212

[32] Schneider MP, Boesen EI, Pollock DM (2007) Contrasting actions of endothelin ET(A) and ET(B) receptors in cardiovascular disease. Annu Rev Pharmacol Toxicol 47: 731–759. 10.1146/annurev.pharmtox.47.120505.105134 17002597 PMC2825895

[33] Shao D, Park JE, Wort SJ (2011) The role of endothelin-1 in the pathogenesis of pulmonary arterial hypertension. Pharmacol Res 63: 504–511. S1043-6618(11)00067-3 [pii]; 10.1016/j.phrs.2011.03.003 21419223

[34] Shimoda LA, Sham JS, Liu Q, Sylvester JT (2002) Acute and chronic hypoxic pulmonary vasoconstriction: a central role for endothelin-1? Respir Physiol Neurobiol 132: 93–106. S1569904802000526 [pii]; 10.1016/s1569-9048(02)00052-6 12126698

[35] Shimoda LA, Laurie SS (2013) Vascular remodeling in pulmonary hypertension. J Mol Med (Berl) 91: 297–309. 10.1007/s00109-013-0998-0 23334338 PMC3584237

[36] Wiklund A, Kylhammar D, Radegran G (2012) Levosimendan attenuates hypoxia-induced pulmonary hypertension in a porcine model. J Cardiovasc Pharmacol 59: 441–449. 10.1097/FJC.0b013e31824938f0 22240915

[37] Ebade AA, Khalil MA, Mohamed AK (2013) Levosimendan is superior to dobutamine as an inodilator in the treatment of pulmonary hypertension for children undergoing cardiac surgery. J Anesth 27: 334–339. 10.1007/s00540-012-1537-9 23223915

[38] Poidinger B, Kotzinger O, Rutzler K, Kleinsasser A, Zierer A, Knotzer H (2019) Intravenous Levosimendan and Vasopressin in New-Onset Acute Pulmonary Hypertension After Weaning from Cardiopulmonary Bypass. J Cardiothorac Vasc Anesth 33: 328–333. S1053-0770(18)30514-7 [pii]; 10.1053/j.jvca.2018.07.013 30122612

[39] Chaliki HP, Hurrell DG, Nishimura RA, Reinke RA, Appleton CP (2002) Pulmonary venous pressure: relationship to pulmonary artery, pulmonary wedge, and left atrial pressure in normal, lightly sedated dogs. Catheter Cardiovasc Interv 56: 432–438. 10.1002/ccd.10203 12112902

[40] Montani D, Price L, Dorfmuller P, Achouh L, Jais X, Yaici A, et al (2009) Pulmonary veno-occlusive disease. Eur Respir J 33: 189–200. 10.1183/09031936.00090608 19118230

[41] Townsley MI (2012) Structure and composition of pulmonary arteries, capillaries, and veins. Compr Physiol 2: 675–709. 10.1002/cphy.c100081 23606929 PMC3630377

[42] Feletou M, Girard V, Canet E (1995) Different involvement of nitric oxide in endothelium-dependent relaxation of porcine pulmonary artery and vein: influence of hypoxia. J Cardiovasc Pharmacol 25: 665–673. 10.1097/00005344-199504000-00022 7596137

[43] Norel X, Walch L, Gascard J, deMontpreville V, Brink C (2004) Prostacyclin release and receptor activation: differential control of human pulmonary venous and arterial tone. Br J Pharmacol 142: 788–796. 10.1038/sj.bjp.0705843 15172959 PMC1575053

[44] Rieg AD, Rossaint R, Uhlig S, Martin C (2011) Cardiovascular agents affect the tone of pulmonary arteries and veins in precision-cut lung slices. PLoS ONE 6: e29698 10.1371/journal.pone.0029698;PONE-D-11-15270 [pii]. 22216346 PMC3246495

[45] Rieg AD, Suleiman S, Anker C, Verjans E, Rossaint R, Uhlig S, et al (2018) PDGF-BB regulates the pulmonary vascular tone: impact of prostaglandins, calcium. Respir Res 19: 120 10.1186/s12931-018-0829-5;10.1186/s12931-018-0829-5 [pii]. 29921306 PMC6009037

[46] Usta C, Eksert B, Golbasi I, Bigat Z, Ozdem SS (2006) The role of potassium channels in the vasodilatory effect of levosimendan in human internal thoracic arteries. Eur J Cardiothorac Surg 30: 329–332. 10.1016/j.ejcts.2006.05.019 16829109

[47] Pataricza J, Hohn J, Petri A, Balogh A, Papp JG (2000) Comparison of the vasorelaxing effect of cromakalim and the new inodilator, levosimendan, in human isolated portal vein. J Pharm Pharmacol 52: 213–217. 10.1211/0022357001773715 10714952

[48] Pataricza J, Krassoi I, Hohn J, Kun A, Papp JG (2003) Functional role of potassium channels in the vasodilating mechanism of levosimendan in porcine isolated coronary artery. Cardiovasc Drugs Ther 17: 115–121. 10.1023/a:1025331617233 12975592

[49] Nijhawan N, Nicolosi AC, Montgomery MW, Aggarwal A, Pagel PS, Warltier DC (1999) Levosimendan enhances cardiac performance after cardiopulmonary bypass: a prospective, randomized placebo-controlled trial. J Cardiovasc Pharmacol 34: 219–228. 10.1097/00005344-199908000-00007 10445673

[50] Gruhn N, Nielsen-Kudsk JE, Theilgaard S, Bang L, Olesen SP, Aldershvile J (1998) Coronary vasorelaxant effect of levosimendan, a new inodilator with calcium-sensitizing properties. J Cardiovasc Pharmacol 31: 741–749. 10.1097/00005344-199805000-00013 9593074

[51] Endoh M (2007) Could Ca2+ sensitizers rescue patients from chronic congestive heart failure? Br J Pharmacol 150: 826–828. 0707163 [pii]; 10.1038/sj.bjp.0707163 17325657 PMC2013876

[52] Rabe KF, Tenor H, Dent G, Schudt C, Nakashima M, Magnussen H (1994) Identification of PDE isozymes in human pulmonary artery and effect of selective PDE inhibitors. Am J Physiol 266: L536–L543. 10.1152/ajplung.1994.266.5.L536 7515580

